# Data on rumen and faeces microbiota profiles of Yakutian and Kalmyk cattle revealed by high-throughput sequencing of 16S rRNA gene amplicons

**DOI:** 10.1016/j.dib.2020.106407

**Published:** 2020-10-11

**Authors:** Vladimir Ya Kataev, Ivan I. Sleptsov, Andrey A. Martynov, Bator K. Aduchiev, Yuri A. Khlopko, Sergey A. Miroshnikov, Sergey V. Cherkasov, Andrey O. Plotnikov

**Affiliations:** a Institute for Cellular and Intracellular Symbiosis of the Ural Branch of the Russian Academy of Sciences, 11 Pionerskaya St., Orenburg 460000, Russian Federation; b Arctic State Agrotechnological University, 15 Krasilnikov St., Yakutsk 677007, Russian Federation; c Federal Research Centre of Biological Systems and Agro-technologies of RAS, 29 9th Yanvarya St., Orenburg 460000, Russian Federation

**Keywords:** Cattle, Yakutian breed, Kalmyk breed, Rumen microbiota, Faeces microbiota, Microbiome, 16S rRNA gene, NGS

## Abstract

It is known that the rumen microbiome directly or indirectly contributes to animal production, and may be a prospective target for mitigation of greenhouse gas emissions [1]. At the same time, feed types and components of diet can influence the composition of the rumen microbiome [2,3]. Fluctuations in the composition of the digestive tract microbiota can alter the development, health, and productivity of cattle [4]. Many studies of cattle microbiomes have focussed on the rumen microbiota, whereas the faecal microbiota has received less attention [5], [6], [7]. Therefore, the features of the faecal and the ruminal microbiomes in different cattle breeds are yet to be studied. Here, we provided 16S rRNA gene amplicon data of the ruminal and the faecal microbiomes from Yakutian and Kalmyk cattle living in the Republic of Sakha, Yakutia, Russia. Total DNA was extracted from 13 faecal and 13 ruminal samples, and DNA libraries were prepared and sequenced on an Illumina MiSeq platform. Paired-end raw reads were processed, and final operational taxonomic units (OTUs) were assigned to the respective prokaryotic taxa using the RDP (Ribosomal Database Project) database. Analysis of the microbiome composition at the phylum level revealed very similar faecal microbiota between the introduced Kalmyk breed and the indigenous Yakutian breed, whereas the ruminal microbiomes of these breeds differed substantially in terms of relative abundance of some prokaryotic phyla. We believe that the data obtained may provide new insights into the dynamics of the ruminal and the faecal microbiota of cattle as well as disclose breed-specific features of ruminal microbiomes. Besides, these data will contribute to our understanding of the ruminal microbiome structure and function, and might be useful for the management of cattle feeding and ruminal methane production.

## Specifications Table

**Table:** 

Subject	Biology
Specific subject area	Metagenomics
Type of data	DNA sequences, table, figures
How data was acquired	16S rRNA gene amplicon sequencing using Illumina MiSeq
Data format	Raw, filtered, and analysed reads
Parameters for data collection	Sampling; isolation of total DNA; library preparation; sequencing; bioinformatic processing and analysis
Description of data collection	Samples of ruminal fluid and faeces were collected from Yakutian and Kalmyk cattle living in the Republic of Sakha (Yakutia), Russia. The faeces were collected with a sterile instrument, while ruminal fluid was obtained by rumenocentesis. Total DNA was isolated using a FastDNA® SPIN Kit for feces. Preparation of the DNA libraries was performed according to the Illumina protocol using primers for the V3–V4 region of the 16S rRNA gene. Paired-end 2 × 300-bp sequencing was carried out on a MiSeq platform. Bioinformatic processing of the raw reads included merging; quality filtering and size selection; evaluation of the filtering quality; OTU formation; removal of chimeras, singletons and doubletons; and taxonomic classification.
Data source location	Institute for Cellular and Intracellular Symbiosis of the Ural Branch of the Russian Academy of Sciences, Orenburg, Russia. Latitude and longitude of sample collection: 62.1104 N, 130.0103 E, settlement Tehtyur, ulus Megino-Kangalassky; 62.1476 N, 128.0588 E, settlement Magaras, ulus Gorny, Republic of Sakha (Yakutia), Russia.
Data accessibility	Raw reads have been deposited at the National centre for Biotechnology Information (NCBI) Sequence Read Archive (Table 1). Additional data related to the design of the experiment are presented in the NCBI BioProject PRJNA627550.

## Value of the Data

 

• This dataset provides a description and comparison of the ruminal and the faecal microbiomes in cattle of Yakutian and Kalmyk breeds based on high-throughput sequencing of 16S rRNA gene amplicons.
• Analysis of 16S rRNA gene sequences at the phylum level revealed very similar faecal microbiota between the introduced Kalmyk breed and the indigenous Yakutian breed as well as breed-specific ruminal microbiome profiles featured by differentially distributed prokaryotic phyla.
• The data on the microbiomes of Kalmyk and Yakutian cattle adapted to cold weather conditions provide insights that would allow to improve livestock rearing in regions with harsh climatic conditions.

## 1 Data Description

**Fig. 1:**
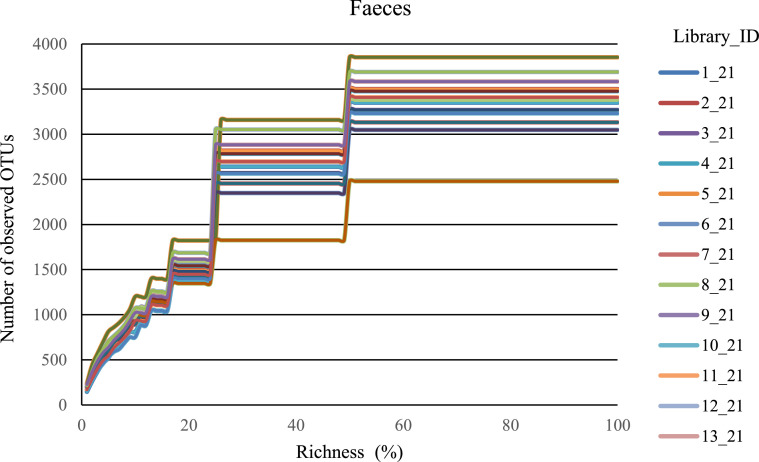
Alpha rarefaction for samples of faeces of Yakutian (1–6) and Kalmyk (7–13) cattle. Number of OTUs with at least one read for each sample.

**Fig. 2:**
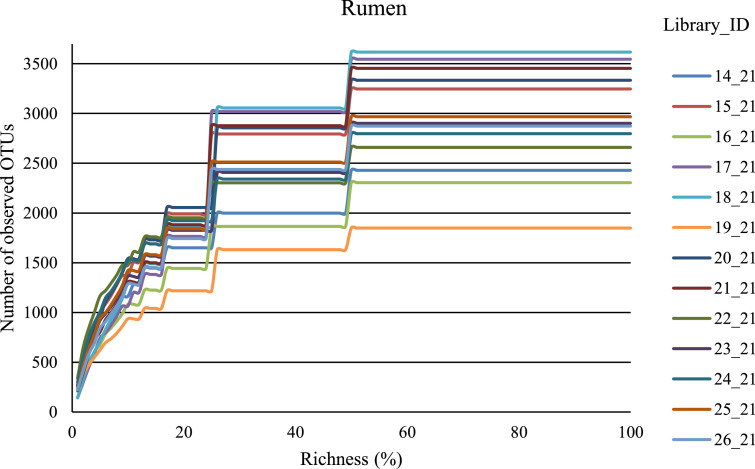
Alpha rarefaction for samples of ruminal fluid of Yakutian (14–19) and Kalmyk (20–26) cattle. Number of OTUs with at least one read for each sample.

The data presented in this article were obtained from samples of rumen and faeces of Yakutian and Kalmyk cattle living in the Republic of Sakha (Yakutia), Russia. Microbiota profiles were revealed by high-throughput sequencing of amplicons containing the V3–V4 region of the 16S rRNA gene. For faecal samples from Yakutian and Kalmyk cattle, a total of 1469,832 raw reads was obtained (mean per sample: 113,064; max. per sample: 150,082; min. per sample, 93,204) (Table 1). After quality and length filtering, 1184,368 reads remained (mean per sample: 91,105; max. per sample: 119,845; min. per sample, 75,201). For ruminal fluid samples of Yakutian and Kalmyk cattle, 1537,066 raw reads were obtained (mean per sample: 118,235; max. per sample: 138,079; min. per sample, 97,016). After filtering, the number of clean reads for this group of libraries decreased to 1226,648 (mean per sample: 94,357; max. per sample: 109,021; min. per sample, 77,716). However, rarefaction plots (Figs. 1, 2) demonstrated that the depth of sequencing was sufficient, as all the curves reached a plateau.

**Table 1:** Sequencing statistics.

Lib_ID	Sample description	SRA accession number	Number of raw reads	Number of cleaned reads	Number of observed OTUs
Experimental group 1					
1_21	Yakutian cow feces	SRR11602067	93,204	75,201	3350
2_21	Yakutian cow feces	SRR11602066	107,646	87,408	3500
3_21	Yakutian cow feces	SRR11602054	108,800	88,259	3268
4_21	Yakutian cow feces	SRR11602048	118,815	94,893	3777
5_21	Yakutian cow feces	SRR11602046	150,082	119,845	3852
6_21	Yakutian cow feces	SRR11602045	107,482	84,773	3048
Experimental group 2					
7_21	Kalmyk cow feces	SRR11602044	108,375	88,330	3132
8_21	Kalmyk cow feces	SRR11602047	95,810	77,603	2480
9_21	Kalmyk cow feces	SRR11602069	122,509	99,785	3375
10_21	Kalmyk cow feces	SRR11602068	106,244	86,196	3230
11_21	Kalmyk cow feces	SRR11602065	103,796	84,073	3409
12_21	Kalmyk cow feces	SRR11602064	123,609	99,136	3690
13_21	Kalmyk cow feces	SRR11602062	123,460	98,766	3585
Experimental group 1					
14_21	Yakutian cow rumen	SRR11602061	123,046	97,567	2428
15_21	Yakutian cow rumen	SRR11602060	120,577	96,080	3247
16_21	Yakutian cow rumen	SRR11602063	114,550	92,329	2304
17_21	Yakutian cow rumen	SRR11602059	109,082	87,943	3547
18_21	Yakutian cow rumen	SRR11602058	104,911	83,579	3617
19_21	Yakutian cow rumen	SRR11602057	130,431	107,801	1849
Experimental group 2					
20_21	Kalmyk cow rumen	SRR11602056	138,079	109,021	3335
21_21	Kalmyk cow rumen	SRR11602053	124,800	98,342	3453
22_21	Kalmyk cow rumen	SRR11602052	129,268	101,359	2660
23_21	Kalmyk cow rumen	SRR11602055	97,016	77,716	2900
24_21	Kalmyk cow rumen	SRR11602051	121,711	96,543	2798
25_21	Kalmyk cow rumen	SRR11602050	109,858	87,789	2968
26_21	Kalmyk cow rumen	SRR11602049	113,737	90,669	2873

**Fig. 3:**
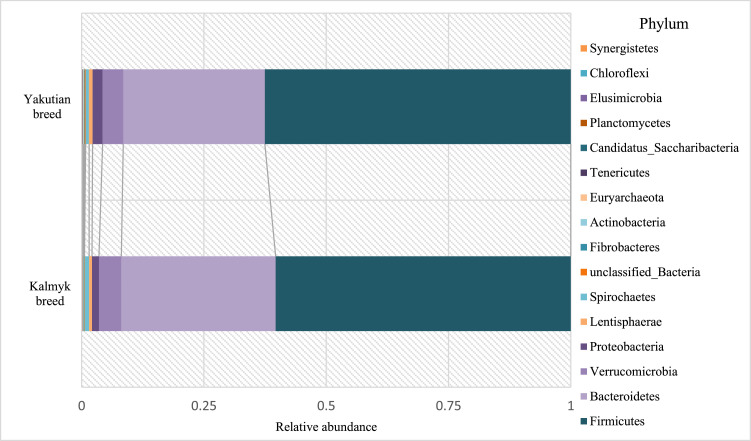
Taxonomic classification of OTUs at the phylum level for faecal microbiomes of the aboriginal Yakutian breed (above) and the introduced Kalmyk breed (below).

The taxonomic classification of OTUs at the phylum level showed that in faecal samples of Yakutian and Kalmyk cattle (Fig. 3), the phyla Firmicutes (62.55% and 60.34%, respectively) and Bacteroidetes (28.93% and 31.52%, respectively) were the most abundant. Conversely, the phyla Verrucomicrobia (4.22% and 4.59%, respectively) and Proteobacteria (2.05% and 1.42%, respectively) were the least abundant. Other phyla, namely, Synergistetes, Chloroflexi, Planctomycetes, Tenericutes, Euryarchaeota, Actinobacteria, Fibrobacteres, Spirochaetes, Lentisphaerae, *Cand.* Saccharibacteria, Elusimicrobia, and unclassified_Bacteria were not numerous and accounted only for 2.25% and 2.14% of the total number of reads for the Yakutian and Kalmyk cattle faecal samples, respectively. Among these, only two phyla demonstrated clear differential abundance, two-fold or more, between Yakutian and Kalmyk cattle faecal samples, namely, *Cand.* Saccharibacteria (0.08% and 0.03%, respectively) and Elusimicrobia (0.02% and 0.01%, respectively).

**Fig. 4:**
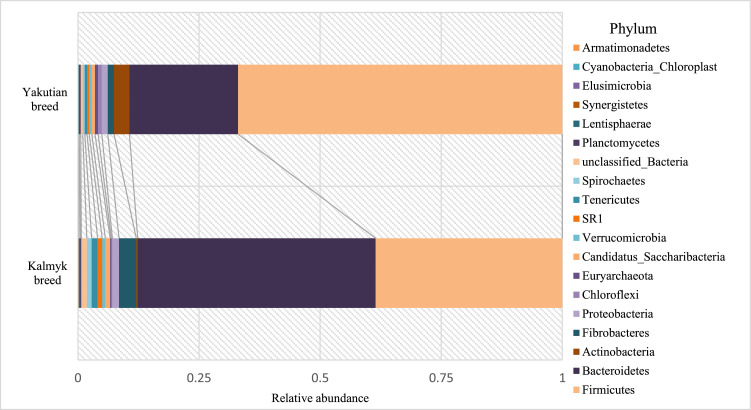
Taxonomic classification of OTUs at the phylum level for ruminal fluid microbiomes of the aboriginal Yakutian breed (above) and the introduced Kalmyk breed (below).

In ruminal fluid samples, the phyla Firmicutes and Bacteroidetes were also predominant, but their relative abundances drastically differed between the Yakutian and the Kalmyk cattle samples. Particularly, the percentages of Firmicutes were 66.91% and 38.54%, respectively, whereas the proportions of Bacteroidetes were 22.46% and 49.11%, respectively (Fig. 4). In the ruminal fluid samples of Yakutian and Kalmyk cattle, there were several poorly abundant phyla, which displayed quite similar abundances, namely, Proteobacteria (1.25% and 1.39%, respectively), *Cand.* Saccharibacteria (0.64% and 0.98%, respectively), Verrucomicrobia (0.53% and 0.68%, respectively), Planctomycetes (0.29% and 0.19%, respectively), Synergistetes (0.06% in both), Cyanobacteria_Chloroplast (0.03% and 0.02%, respectively), and Armatimonadetes (0.02% in both). At the same time, the relative abundances of other phyla differed sharply, two times or more, between Yakutian and Kalmyk cattle ruminal fluid samples. Particularly, the following phyla demonstrated differential abundance in the ruminal fluid samples of Yakutian and Kalmyk cattle: Actinobacteria (3.18% and 0.30%, respectively), Fibrobacteres (1.27% and 3.55%, respectively), Chloroflexi (0.74% and 0.22%, respectively), SR1 (0.47% and 0.96%, respectively), Tenericutes (0.44% and 1.20%, respectively), Spirochaetes (0.43% and 1.0%, respectively), Lentisphaerae (0.15% and 0.32%, respectively), Euryarchaeota (0.66% and 0.23%, respectively), unclassified_Bacteria (0.42% and 1.16%, respectively), and Elusimicrobia (0.03% and 0.08%, respectively).

## 2 Experimental Design, Materials and Methods

### 2.1 Experimental design

The aim of this study was to assess the composition of rumen and faeces microbiomes in cattle of the introduced Kalmyk breed and the indigenous Yakutian breed. The composition of both groups of Kalmyk (*n* = 7) and Yakutian (*n* = 6) cattle was optimised for sex (cows only), age (4–7 years old), and weight (350–480 kg). Animals in both groups were kept under similar conditions and provided the same feed rations.

### 2.2 Sample collection

Samples of faeces and rumen fluid were collected from cattle of the Yakutian and Kalmyk breeds in the Republic of Sakha (Yakutia), Russia, in October 2019. The cattle of Yakutian breed were kept on the farm Kylys (62.1104 N, 130.0103 E), settlement Magaras, ulus Gorny. The cattle of Kalmyk breed were kept on the farm Soloosun (62.1476 N, 128.0588 E), settlement Tehtyur, ulus Megino-Kangalassky.

Faecal samples were obtained from the selected cows by a non-invasive method. After defecation, the top layer of the faeces was removed with a sterile spatula, and then 0.4–0.5 g of faeces was transferred into a 2.0-mL Eppendorf tube containing 500 µL of a DNA/RNA Shield (Zymo Research, Irvine, CA, USA) preservative solution. Samples of ruminal fluid were obtained by rumenocentesis with a sterile needle under local anaesthesia by observing the rules of an aseptic technique. Afterwards, 0.5 mL of ruminal fluid was transferred into a 2.0-mL Eppendorf tube containing 500 µL of DNA/RNA Shield.

Sampling was carried out on the same day for all animals of the same group. The samples were transported to the laboratory at 4–25 °C in accordance with the manual of the DNA/RNA Shield preservative.

### 2.3 DNA extraction and 16S rRNA gene sequencing

Total DNA from ruminal fluid or faeces was isolated using a FastDNA® SPIN Kit for Faeces (MP Biomedicals Inc., Solon, OH, USA) by applying a Lysing Matrix E. Samples were homogenised on a TissueLyser LT (Qiagen, Venlo, Netherlands). The duration of homogenisation was increased up to 5 min, in contrast to the manufacturer's protocol. The quality of the extracted DNA was assessed by electrophoresis in 1% agarose gel and with Nanodrop 8000 (Thermo Fisher Scientific, Inc., Waltham, MA, USA). The DNA concentration was quantified using a Qubit 4.0 Fluorometer with a dsDNA High Sensitivity Assay Kit (Life Technologies, Carlsbad, CA, USA).

DNA libraries were prepared according to the Illumina two-step protocol (Part #15,044,223, Rev. B). At the first stage, target amplicons were prepared using primers for the V3–V4 region of the 16S rRNA gene (S-D-Bact-0341-b-S-17 and S-D-Bact-0785-a-A-21) [8], which were connected to Illumina overhang sequences. The composition of the PCR mixture and the PCR parameters are presented in Table 2. At the second stage, the amplicons were bound with sample-specific dual Illumina indices (Nextera XT, i7 and i5). Paired-end 2 × 300-bp sequencing was carried out on an MiSeq platform ((Illumina, San Diego, CA, USA) with a Reagent Kit v.3 (Illumina).

**Table 2:** Composition of PCR mixture and parameters of PCR.

Components of PCR mixture (25 µl)	Final content	PCR parameters: 98°С, 1 min (initial denaturation) 25 cycles 98°С, 10 s (denaturation) 56°С, 30 s (annealing) 72°С, 30 s (extension) 72°С, 2 min (final extension)
Template DNA	25 ng	
Forward and reverse primers	0,2 µM each	
dNTPs	200 µM each	
Q5 High-Fidelity DNA Polymerase	0,5 U	
5X Q5 Reaction Buffer	1Х	
Nuclease-free water	until 25 µl	

### 2.4 Bioinformatics and statistical analysis

Paired-end reads were merged with a minimal overlap of 40 bp and a p-value of 0.0001 using PEAR v. 0.9.10 [9]. Subsequent treatment of the merged reads was conducted with USEARCH v. 10.0.240 [10] and included quality filtering and amplicon size selection (minimal size, 420 bp). Reads shorter than 420 bp and reads with an expected error (ee) higher than 1 per 100 nucleotides (max. ee, 1.0) were filtered out. Filtering quality was evaluated using FastQC v. 0.11.7. Due to dereplication and clustering with USEARCH, OTUs were formed, whereas singletons and doubletons were removed. OTUs were determined using a similarity threshold level of 97% between sequences to classify microorganisms at the species level. Chimeric sequences were detected and removed using USEARCH via UCHIME [11]. Contaminant OTUs were identified and removed via the USEARCH command ‘ublast’ by matching the sequences of trial samples and negative control samples. The taxonomic classification of sequences was conducted using the RDP [12] and NCBI reference databases. Rarefaction curves were built using Microsoft Office Excel, based on the data obtained with the ‘alpha_div_rare’ command (USEARCH v.11).

## CRediT Author Statement

**Vladimir Ya. Kataev:** Investigation, Validation, Writing - Original Draft, Writing - Review & Editing. **Ivan I. Sleptsov:** Methodology, Investigation, Resources, Writing - Original Draft. **Andrey A. Martynov:** Sampling, Investigation, Resources, Writing - Original Draft. **Bator K. Aduchiev:** Sampling, Investigation, Resources. **Yuri A. Khlopko:** Software, Formal analysis, Data Curation. **Sergey V. Miroshnikov:** Supervision, Funding acquisition, Writing - Review & Editing. **Sergey V. Cherkasov:** Supervision, Resources, Writing - Review & Editing. **Andrey O. Plotnikov:** Methodology, Writing - Original Draft, Writing - Review & Editing.

## Ethical Statement

This sampling was carried out in accordance with the recommendations of the National Institutes of Health Guide for the Care and Use of Laboratory Animals (NIH Publications No. 8023, revised 1978).

## Declaration of Competing Interest

The authors declare that they have no known competing financial interests or personal relationships that could have appeared to influence the work reported in this paper.
